# The role of amygdala reactivity in affective fluctuations across social contexts

**DOI:** 10.1038/s41598-025-22131-x

**Published:** 2025-10-31

**Authors:** Chae-eun Chung, Hakin Kim, Junhyun Park, M. Justin Kim, Juyoen Hur

**Affiliations:** 1 Department of Psychology, Yonsei University, Seoul, 03722 Republic of Korea; 2 Department of Psychology, Sungkyunkwan University, Seoul, 03063 Republic of Korea; 3 Center for Neuroscience Imaging Research, Institute for Basic Science, Suwon, 16419 Republic of Korea

**Keywords:** Amygdala, Positive and negative affect, Social contexts, FMRI, Ecological momentary assessment, Psychology, Amygdala, Neuroscience, Emotion, Social neuroscience

## Abstract

The amygdala plays a critical role in socio-emotional processing, serving not only as a key neural substrate for shaping emotional experiences and social behavior, but also as a trait-like individual risk factor that confers heightened vulnerability to emotional disorders, including anxiety and depression. While prior research has primarily examined the link between heightened amygdala reactivity and negative affect (NA), much less is known about its relationship with positive affect (PA), particularly across different social contexts. In this study, we focused on how these associations vary based on the level of social intimacy and distinct facets of affect (e.g., high vs. low-arousal PA). Using a combined fMRI and ecological momentary assessment (EMA) approach, we examined how individual differences in amygdala reactivity relate to momentary emotional experiences across diverse social contexts in daily life. As expected, interactions with close companions had a robust mood-enhancing effect. Notably, individual differences in amygdala reactivity moderated the association between social context and high-arousal PA, such that individuals with heightened amygdala reactivity reported lower levels of high-arousal PA when alone, compared to when they were with close others. These findings highlight the specific social contexts and affective states most relevant to individual differences in amygdala reactivity, offering novel insights into the dynamic interplay between brain function, emotional experiences, and social contexts.

**Supplementary Information:**

The online version contains supplementary material available at 10.1038/s41598-025-22131-x.

## Introduction

The amygdala is a central brain structure involved in socio-emotional processing, playing a pivotal role in emotion experiences and social behavior^1–3^. Aberrant amygdala reactivity, characterized by exaggerated responses to negative emotional stimuli, has been consistently linked to increased stress reactivity and impaired social functioning, underscoring its critical role in mental health and well-being within social contexts^4–6^. More recently, individual differences in amygdala reactivity have been identified as a potential biomarker of vulnerability to emotional disorders, such as anxiety and depression, particularly when coupled with exposure to aversive life events^7–10^. Despite its significance, relatively little is known about how amygdala reactivity – conceptualized as a trait-like individual risk factor for emotional distress and dysfunction – relates to everyday emotional experiences, especially particularly with respect to its contextual specificity across diverse real-world social contexts.

Research on the relationship between amygdala reactivity and socio-emotional processes has predominantly focused on negative affect (NA), such as feelings of rejection, anxiety, and loneliness^11–13^. For example, individuals with heightened amygdala reactivity to fearful faces are more likely to report frequent and intense experiences of social humiliation^11^, and elevated separation anxiety^12^. These findings suggest that heightened amygdala reactivity may increase susceptibility to elevated NA in social contexts. In contrast, relatively little is known about whether heightened amygdala reactivity also serves as a neural risk factor in the domain of positive affect (PA), as reflected in a reduced capacity to experience PA. Only a few studies have examined this question: one found that individuals with heightened amygdala responses to unpleasant stimuli reported lower levels of trait PA^14^, and another showed that a more persistent pattern of amygdala activation to aversive images was associated with lower overall PA^6^. Although these findings suggest a potential link between heightened amygdala reactivity and diminished PA, prior work has primarily focused on trait-level affect or general mood, limiting insight into how individual differences in amygdala reactivity relate to momentary fluctuations in PA across diverse social contexts. Given that difficulty sustaining PA – alongside elevated NA, represents a distinct and clinically relevant phenotype of emotional disturbance and internalizing psychopathology^15,16^, addressing this gap is crucial.

Social engagement is generally associated with beneficial affective outcomes, such as decreased NA and increased PA^17–20^. For example, individuals undergoing stress-inducing tasks exhibit improved mood when accompanied by close friends or romantic partners^21,22^. Moreover, the intensity and pattern of these mood-lifting effects likely vary depending on the intimacy of the social connection. Granovetter^23^ distinguished between ‘strong’ and ‘weak’ social ties, differing in the level of intimacy with the interaction partner. While interactions with close connections (e.g., close friends, romantic partners) reliably reduce NA^24^ and increase PA^19^, the effects of interactions with weaker ties (e.g., co-workers, acquaintances) on affective experiences are less clear^20,25–27^. Thus, the present study not only investigated the relationship between amygdala reactivity and mood, but also explored the role of social contexts with varying levels of intimacy.

The results of previous research indicate that to understand the link between social contexts and PA, it is important to distinguish between high-arousal and low-arousal PA^28,29^. Berenbaum^30^ found that the emotional benefits of social interaction were particularly pronounced for high-arousal PA, such as cheerfulness and joy, but not for low-arousal positive states, such as contentment and calmness. Some studies have even reported that low-arousal PA may decrease during interactions with friends^31,32^. These findings suggest the need to investigate whether the mood-lifting effects of social engagement are specific to high-arousal PA and how these effects are influenced by social contexts and individual differences in amygdala reactivity.

Most existing research relies on retrospective self-reports or controlled laboratory settings, limiting the ecological validity of the findings^11–13^. Ecological momentary assessment (EMA) is a powerful tool for capturing participants’ real-time emotional experiences across diverse real-world contexts. EMA not only minimizes biases and distortions associated with retrospective reports^33–35^ but also mitigates ecological fallacy—errors arising from drawing conclusions about individuals based on aggregated group data^36^. Moreover, EMA provides unique insights into the nuanced real-world dynamics of affective experiences (e.g., high vs. low-arousal PA vs. NA) in various social contexts.

This study integrated brain imaging and smartphone-based EMA to examine how amygdala reactivity, conceptualized as a potential risk factor for emotional distress and dysfunction relates to fluctuations in emotional experiences across diverse social contexts in daily life. To our knowledge, this is the first fMRI-EMA fusion study to investigate the relationship between amygdala reactivity and PA, with a specific focus on high- vs. low-arousal PA. Given the pivotal role of social context in emotional experiences, these relationships were examined within different social contexts varying in levels of intimacy. Based on prior research, we hypothesized an overall mood-lifting effect in the presence of others – specifically, increased high- and low-arousal PA and decreased NA – with the strongest effects expected when individuals were with close companions, compared to distant others (i.e. weak ties). Additionally, given that past research has demonstrated that high-arousal PA is more sensitive to social interaction than is low-arousal PA^30,37^, we predicted that amygdala reactivity would be associated with fluctuations in high-arousal PA, but not low-arousal PA.

## Method

### Overview

All participants were healthy Korean young adults under 25 years old who had consistent access to a smartphone for EMA. Participants reported the absence of current internalizing disorders (e.g., major depressive disorder, generalized anxiety disorder, panic disorder, social anxiety disorder), suicidal ideation, psychiatric treatment, and psychiatric medicine-taking (See details in Supplementary Materials). All participants also reported the absence of lifetime neurological disorders, alcohol/substance abuse, or MRI contraindications. At the baseline laboratory session, participants were familiarized with the EMA protocol. Beginning the next day, participants completed up to five EMA surveys/day for two weeks and completed the neuroimaging assessment as well. All procedures were approved by the Institutional Review Board (IRB) of Yonsei University (Seoul, South Korea), and all participants provided written informed consent. All methods were performed in accordance with the relevant guidelines and regulations. The sample overlaps that featured in prior work by our group focused on neuroticism^38^.

### Participants

A total of 116 participants completed both the EMA protocol and fMRI assessment. The target sample size was determined to ensure acceptable power and precision^39,40^ while accounting for resource constraints. G*Power^41^ indicated that a total sample size of *N* = 116, with an alpha level of 0.05 (two-tailed) and an assumed medium effect size (*r* = 0.30), yielded an estimated power of 0.92, indicating sufficient sensitivity to detect effects of this magnitude. Of these participants, 9 participants were excluded from EMA analyses because of their low response rate (less than 50% responses). Of these 107 participants with usable EMA data, 23 participants were excluded from fMRI analyses because of excessive movement in the scanner, defined as scans with motion outliers (i.e., framewise displacement > 0.5 mm) exceeded 10% of total volumes (*n* = 8), technical issues (*n* = 11), anatomical brain lesion (*n* = 2), low task compliance (*n* = 1), and distortion correction error (*n* = 1). The final sample included 84 participants (79.8% female; *M* = 22.2 years, *SD* = 2.0 years).

### EMA procedures

#### Protocol

Text messages containing a link to secure online EMA survey were delivered via smartphones five times a day for two weeks. Messages were delivered pseudo-randomly within 12 h of a participant’s preferred time range between 7 a.m. and 11 p.m. Participants were instructed to respond within 15 min and to refrain from responding at unsafe or inconvenient moments (e.g., driving). Several procedures were used to promote compliance, including providing monetary bonuses for increased compliance, and reminding of their response rate on the second and seventh days. In the final sample, EMA compliance was acceptable (*M* = 75.7%, *SD* = 10.7%, Total = 4,448).

#### EMA survey and data reduction

Participants reported their current mood by rating high-arousal PA (*enthusiastic*,* joyful*,* cheerful*), low-arousal PA (*calm*,* content*,* relaxed*), and NA (*nervous*,* worry*,* afraid*,* sad*,* hopeless*,* downhearted*,* irritable*,* angry*,* tired*,* lonely*) items on a 5-point scale (1 = not at all, 5 = extremely). Exploratory factor analysis (EFA) yielded a three-factor solution at the between-subject level, supporting the distinction of mood items into three distinct subscales (See Table S1 in Supplementary Materials). Composite scores for high-arousal PA, low-arousal PA, and NA were calculated by averaging the corresponding items, with all subscales demonstrating high internal consistency ($\:\alpha\:s$ > 0.87).

Participants also indicated their current social context. First, their social interaction status was assessed using a binary item (“Have you been alone since the previous prompt?”). If participants indicated that they were not alone, they were asked who they were with: close person/people (e.g., family, close friends), acquaintance(s) (e.g., co-worker, friends of acquaintance) or stranger(s). Consistent with prior works^42,43^ on social interaction, close person/people were re-coded as ‘close’ companions, and acquaintance(s) and stranger(s) were re-coded as ‘distant’ companions. This approach was to distinguish between strong and weak social connections.

**Fig. 1:**
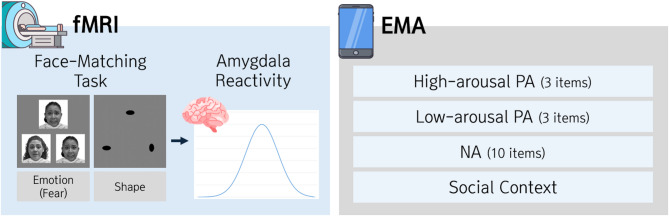
Study overview. All participants were assessed using functional MRI (fMRI), a well-established face-matching task, and ecological momentary assessment (EMA). For the face-matching task, analyses focused on a composite measure of bilateral amygdala reactivity. Smartphone EMA was used to sample momentary fluctuations in negative and positive affect, as well as social contexts (5 surveys/day x 14 days). To integrate the fMRI and EMA data streams, a series of multilevel models (MLMs) was implemented. Abbreviations—PA, positive affect; NA, negative affect.

### fMRI task

#### Face-matching task

Participants performed the modified face-matching task^44^ inside the scanner. The choice of paradigm was rooted in work demonstrating that the amygdala is robustly activated by emotional faces, particularly those depicting expressions of fear^44,45^. This task demonstrates moderate test-retest reliability^46,47^ and has been widely used to assess amygdala reactivity as a biomarker in large-scale studies, including the Duke Neurogenetics Study, IMAGEN, ABCD, and the UK Biobank^48,49^. During this task, a target face appeared at the top center of the screen, while two faces were displayed on both sides at the bottom. Participants were instructed to promptly and accurately select the face at the bottom that matched the target (Fig. 1; see Fig. S1 in Supplementary Materials). The experiment consisted of 16 blocks in a block design: fear (*n* = 2), happy (*n* = 2), surprise (*n* = 2), neutral (*n* = 2), and shape blocks (*n* = 8). The shape condition served as a sensorimotor baseline. Each block lasted 24 s and included six stimuli (3 s each) with a 1-second ISI (Supplementary Fig. S1). Block and trial order were pseudorandomized across participants. To capture robust individual differences in amygdala reactivity, analyses focused on the fear vs. shape contrast (see *fMRI Modeling* section for details). Face stimuli were selected from the Racially Diverse Affective Expression (RADIATE) facial stimulus set^50^, with the number of stimuli balanced across gender and race. Task was presented using E-prime 3.0.

### MRI data acquisition

MRI data were acquired using a Siemens MAGNETOM VIDA 3-tesla scanner with a 32-channel phased array coil. T1-weighted anatomical images were acquired using a magnetization-prepared rapid-acquisition gradient echo (MPRAGE) sequence (TR = 2,300 ms, TE = 2.26 ms, flip angle = 8 degrees, slice thickness = 1 mm, voxel size = 1 × 1 × 1 mm, matrix = 256 × 256, field of view = 256 mm).

To enhance resolution, we used multiband sequence to collect EPI volumes (multiband acceleration = 3, TR = 1,500 ms, TE = 30.00 ms, flip angle = 80 degrees, slice thickness = 2 mm, voxel size = 2 × 2 × 2 mm, matrix = 110 × 110, field of view = 220 mm, transversal slices = 69, volumes = 290). To enable fieldmap correction, 10 spin echo EPI volumes as the same location and resolution as the functional volumes were acquired in opposing direction; posterior to anterior. Additionally, we acquired a double echo gradient EPI image (TR = 672 ms, TE1 = 4.92 ms, TE2 = 7.38 ms, flip angle = 60 degrees, slice thickness = 2 mm, voxel size = 2 × 2 × 2 mm, matrix = 110 × 110, field of view = 220 mm, transversal slices = 69) to generate two magnitude images and a single phase different images.

### MRI data preprocessing

Neuroimaging data were preprocessed using *fMRIPrep 22.1.1*^51^, which is based on *Nipype 1.8.5*^52^. Structural MRI and fMRI data were visually inspected before and after processing for quality assurance.

###  Functional data

EPI files were slice-time corrected using *3dTshift* from *AFNI*^53^. To correct head motion, motion parameters with respect to the BOLD reference were estimated using *mcflirt*^54^. The BOLD references were co-registered to the T1-weighted reference using boundary-based registration^55^. This step was configured with six degrees of freedom. The BOLD time-series were resampled into standard space, generating a preprocessed BOLD run in MNI152 space. Automatic removal of motion artifacts using *ICA-AROMA*^56^ was performed on the preprocessed BOLD and MNI152NLin6Asym space time-series after removal of non-steady state volumes. Spatial smoothing was implemented with an isotropic, Gaussian kernel of 6 mm full width half-maximum (FWHM). Corresponding “non-aggressively” denoised runs were produced after such smoothing.

### fMRI modeling and data reduction

The preprocessed images were analyzed using statistical parametric mapping (SPM12) software (https://www.fil.ion.ucl.ac.uk/spm/). At the subject level, a separate predictor was entered for fear face, happy face, surprise face, neutral face and shape conditions and convolved with a canonical hemodynamic response function (HRF). The temporal high-pass filter was set at a cutoff frequency of 128 s. Additional nuisance variables included non-steady state outliers. For consistency and comparability across analyses of high-arousal PA, low-arousal PA, and NA, we used a single, unified contrast (i.e., Fear > Shape). This contrast was selected for its robust and consistent engagement of the amygdala and its widespread use as a biomarker of emotional reactivity and vulnerability to anxiety and depression^7,44^. While the Happy > Shape contrast can also elicit amygdala activation, its signal is typically weaker and less reliable^57–59^. Anatomically defined region of interest (ROI) masks for the bilateral amygdala were created using FSL Harvard-Oxford atlas (see Fig. S2 and Fig. S3 in Supplementary Materials). Mean contrast values were separately extracted for the left and right amygdala ($\:\alpha\:s$ = 0.91).

### Analytic strategy

The overarching aim of this study was to investigate how amygdala reactivity relates to fluctuations in different facets of emotional experiences (high-arousal PA, low-arousal PA, and NA) across diverse social contexts varying in levels of intimacy in real-world settings. A series of multilevel models (MLMs) was implemented using R software (version 4.3.1) with the *lme4*^60^ and *lmerTest*^61^ packages. In these models, momentary assessments of affect and social context were nested within subjects as Level-1 variables, while intercepts were allowed to vary across subjects. Individuals’ levels of amygdala reactivity were grand-mean centered and included as continuous Level-2 variables. Unlike traditional repeated measures GLM approaches, MLM is well-suited for handling the nested data structure and varying numbers of longitudinal assessments across participants. The restricted maximum likelihood (REML) method was employed for all MLM analyses.

The equations below outline the basic structure of our MLMs in standard notation^62^. At Level 1, momentary affect at time $\:t$ was modeled as a function of social context for individual $\:i.$ Social context was entered as a categorical variable, with “Alone” as the reference category in the primary analyses, and “Distant companions” in follow-up analyses:

#### Level-1 (within-person)

$$\:{\text{A}\text{f}\text{f}\text{e}\text{c}\text{t}}_{\text{t}\text{i}}=\:{\pi\:}_{0i}+\:{\pi\:}_{1i}\left(\text{D}\text{i}\text{s}\text{t}\text{a}\text{n}\text{t}\right)+\:{\pi\:}_{2i}\left(\text{C}\text{l}\text{o}\text{s}\text{e}\right)+{e}_{ti}$$

#### Level-2 (between-person)

$$\:{\pi\:}_{0i}={\:\beta\:}_{00}+\:{r}_{0i}$$

$$\:{\pi\:}_{1i}={\:\beta\:}_{10}+\:{r}_{1i}$$

$$\:{\pi\:}_{2i}={\:\beta\:}_{20}+\:{r}_{2i}$$

To test whether the association between social context and affect was moderated by individual differences in amygdala reactivity, we expanded the Level-2 equations as follows:

#### Level-2 (moderation)

$$\:{\pi\:}_{0i}={\:\beta\:}_{00}+\:{\:\beta\:}_{01}\left({Amygdala\:Reactivity}_{i}\right)+{r}_{0i}$$

$$\:{\pi\:}_{1i}={\:\beta\:}_{10}+\:{\:\beta\:}_{11}\left({Amygdala\:Reactivity}_{i}\right)+{r}_{1i}$$

$$\:{\pi\:}_{2i}={\:\beta\:}_{20}+\:{\:\beta\:}_{21}\left({Amygdala\:Reactivity}_{i}\right)+{r}_{2i}$$

Conceptually, this approach allowed us to examine the cross-sectional relationships between EMA-derived measures of social context and affect, the relationship between amygdala reactivity and affect, and the potential moderating role of amygdala reactivity in the link between social context and affect.

For significant results, we additionally explored the impact of incorporating variation in the amount of time allocated to different contexts as a nuisance variable to confirm that the observed effects are above and beyond that explained by these factors, underscoring the unique explanatory contribution (incremental validity) of the neuroimaging metric (here, amygdala reactivity).

## Results

**Fig. 2:**
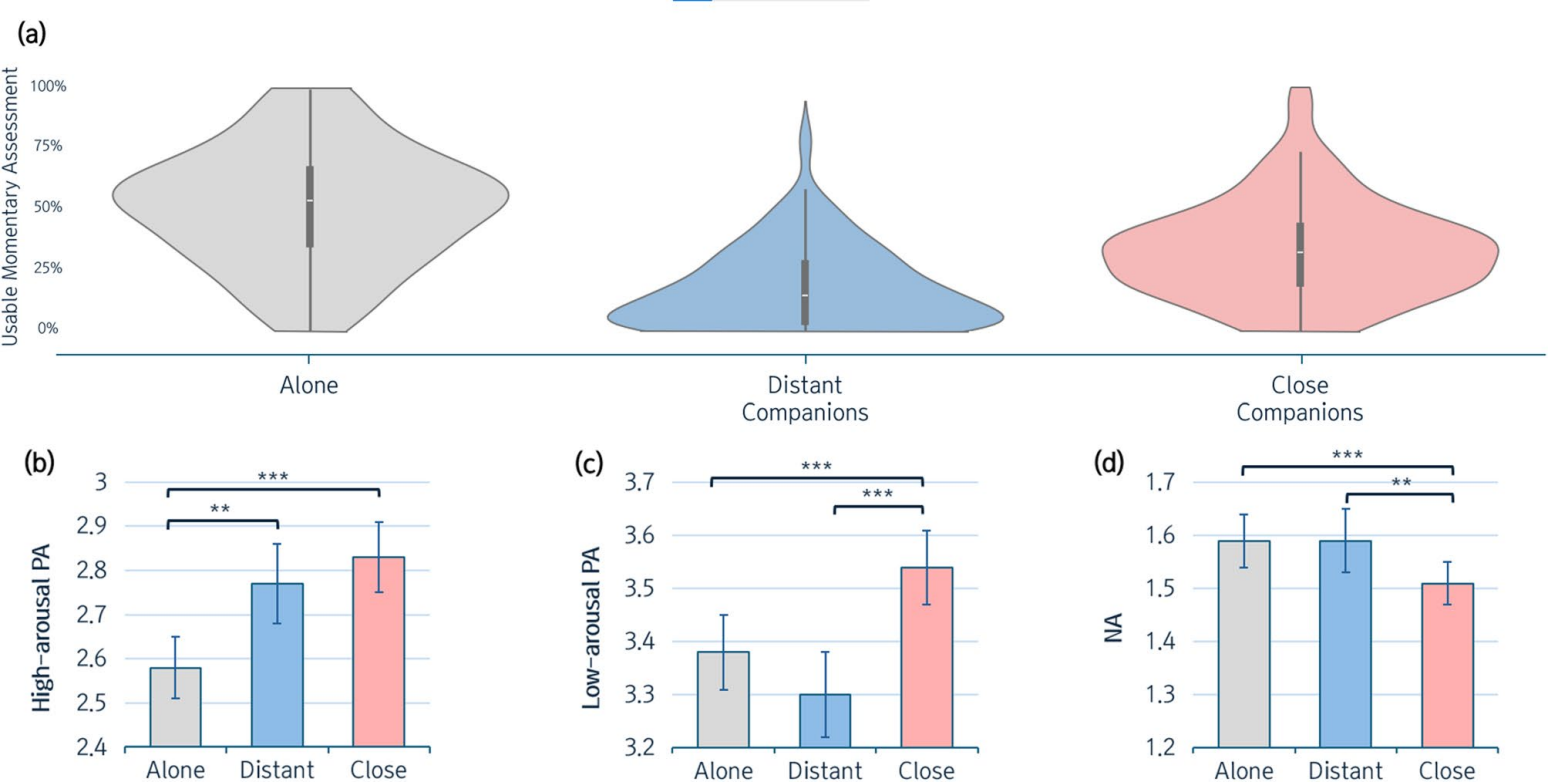
Affective experiences in different social contexts. Panel (**a**) depicts the percentage of usable momentary assessments collected across various social contexts. Panels (**b**–**d**) show the levels of high-arousal PA, low-arousal PA, and NA, respectively, across different social contexts.

Consistent with other research with young adults (Hur et al., 2020; Shackman et al., 2018), participants spent around half of their time with others, although there were marked individual differences in the amount of time devoted to each social context (Alone = 50.75%, Close = 32.82%, Distant = 16.43%) (Fig. 2a). As noted below, the relationships between amygdala activity, social context, and emotional experiences remained consistent even after accounting for the amount of time individuals reported spending in different social contexts.

### Momentary emotional experience in different social contexts

Consistent with prior studies^17,19,63^, the overall mood-lifting effect of social engagement with close others was confirmed. Participants reported significantly higher levels of both high- and low-arousal PA and lower levels of NA when in the presence of close companions compared to being alone (high-arousal PA: *t* = 7.03, *b* = 0.26, *S.E.* = 0.04, *p* < 0.001; low-arousal PA: *t* = 4.36, *b* = 0.15, *S.E.* = 0.04, *p* < 0.001; NA: *t* = −3.65, *b* = −0.08, *S.E.* = 0.02, *p* < 0.001). Though to a lesser degree, social engagement with weak ties also had a significant mood-lifting effect on high-arousal PA (*t* = 3.44, *b* = 0.19, *S.E.* = 0.07, *p* = 0.001; Fig. 2b), but showed no significant effects on low-arousal PA or NA (*p*s > 0.1; Fig. 2c-d). These results remained consistent after controlling for age, sex, and time spent in each social context (see Table S2 and Table S3 in Supplementary Materials).

### Amygdala reactivity, social contexts, and momentary emotional experience

**Fig. 3:**
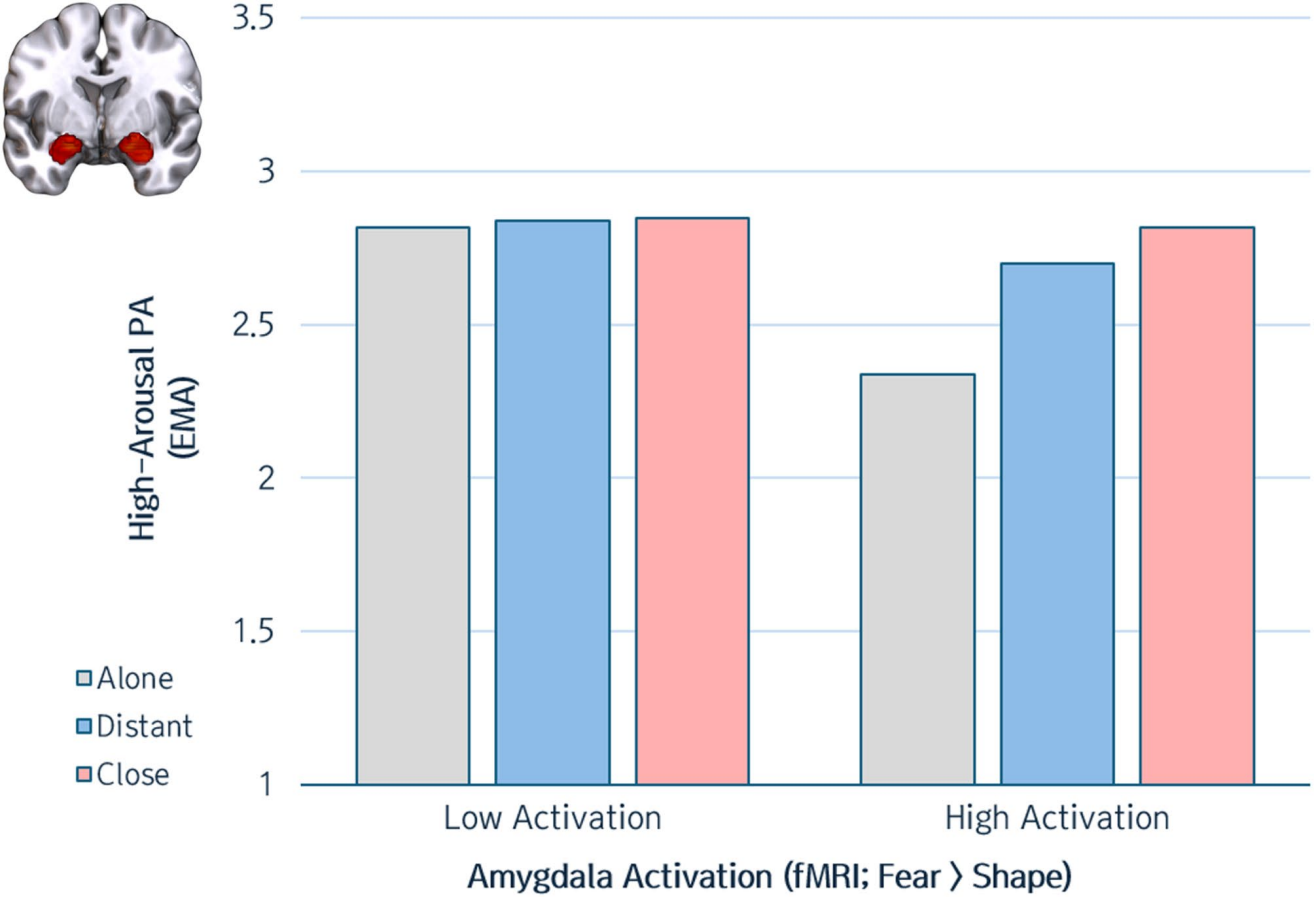
Amygdala reactivity modulated high-arousal PA in different social contexts. As shown in the bar plot, individuals with high amygdala reactivity showed significantly lower levels of high-arousal PA when alone compared to when they were with close others. Individuals with low amygdala reactivity did not show such a pattern. Hypothesis testing relied on a continuous measure of amygdala reactivity. For illustrative purposes, predicted values derived from MLM are depicted for extreme levels (low = −1 *SD*; high = + 1 *SD*) of amygdala reactivity. Brain images were generated using MRIcroGL software (version 1.2.20220720), available at https://www.nitrc.org/projects/mricrogl.

Consistent with our a priori hypothesis, individuals with high amygdala reactivity exhibited greater fluctuations in high-arousal PA across social contexts involving varying levels of intimacy (Table 1). Specifically, as shown in Fig. 3, individuals with high amygdala reactivity reported significantly lower high-arousal PA when alone compared to when they were with close companions (Brain x Close vs. Alone: *t* = 2.01, *b* = 0.22, *S.E.* = 0.11, *p* = 0.047). Although the difference between being with distant companions and being alone was not significant (*t* = 1.09, *b* = 0.17, S.E. = 0.16, *p* = 0.281), a linear trend was observed, with high-arousal PA decreasing as the intimacy of social contexts diminished (alone < distant < close). Conversely, individuals with low amygdala reactivity showed no significant changes in high-arousal PA across social contexts. In contrast, no significant interaction effects between amygdala reactivity and social context were found for momentary low-arousal PA (Close vs. Alone: *t* = 0.41, *b* = 0.05, *S.E.* = 0.11, *p* = 0.68; Distant vs. Alone: *t* = 1.20, *b* = 0.19, *S.E.* = 0.16, *p* = 0.23) or NA (Close vs. Alone: *t* = −0.32, *b* = −0.02, *S.E.* = 0.07, *p* = 0.75; Distant vs. Alone: *t* = 0.66, *b* = 0.06, *S.E.* = 0.09, *p* = 0.51). Furthermore, amygdala reactivity was not directly associated with real-world emotional experiences (high-arousal PA: *t* = −1.06, *b* = −0.24, *S.E.* = 0.23, *p* = 0.29; low-arousal PA: *t* = −1.38, *b* = −0.30, *S.E.* = 0.22, *p* = 0.17; NA: *t* = −0.26, *b* = −0.04, *S.E.* = 0.14, *p* = 0.795). These results remained consistent after controlling age, sex, and the amount of time allocated to social contexts, confirming the unique explanatory value of amygdala reactivity in understanding real-world emotional dynamics (see Table S4 in Supplementary Materials for more details). Although our analyses were guided by an a priori hypothesis focusing on the moderating effect of amygdala reactivity on high-arousal PA, it is worth noting that the observed interaction between amygdala reactivity and close social context on high-arousal PA (*p* = 0.047) did not remain statistically significant after Bonferroni correction for multiple comparisons across the three affective outcomes (adjusted *p* = 0.14). Therefore, this result should be interpreted with caution.

**Table 1:** The joint impact of amygdala reactivity and social contexts on momentary emotional experience in the real world.

	High-arousal PA		Low-arousal PA		NA	
	b	t	b	t	b	t
Amygdala	−0.24	−1.06	−0.27	−1.36	−0.04	−0.26
Close (vs. alone)	0.26	7.23 ***	0.17	3.98 ***	−0.07	−2.38 *
Distant (vs. alone)	0.19	3.50 ***	0.00	0.01	−0.01	−0.31
Amygdala × close (vs. alone)	0.23	2.01 *	0.13	1.40	−0.02	−0.32
Amygdala × distant (vs. alone)	0.17	1.09	0.18	1.31	0.06	0.66

## Discussion

Given the amygdala’s central role in socio-emotional functioning, this study combined fMRI and EMA data to examine how laboratory-based amygdala reactivity – conceptualized as a trait-like individual risk factor for emotional distress and dysfunction – relates to emotional experiences across various social contexts in daily life. Consistent with hypotheses, the results confirmed the robust mood-enhancing effect of interactions with “close” others and revealed that individual differences in amygdala reactivity were particularly related to high-arousal PA in these contexts. Specifically, individuals with high amygdala reactivity reported significantly lower levels of high-arousal PA (e.g., joyful, cheerful, enthusiastic) when alone, compared to when they were with close companions. The implications of these findings are discussed below.

Consistent with prior studies^19,24^, our findings confirmed the mood-lifting effect of close companions across all three facets of affective experience (i.e., high- and low-arousal PA, NA), highlighting the critical role of intimacy in enhancing mood. Although to a lesser extent, social engagement with weak ties (e.g., acquaintances, co-workers) also had a significant mood-lifting effect on high-arousal PA, but showed no significant effects on low-arousal PA or NA. Previous research has yielded mixed findings regarding the emotional impact of weak ties, with some studies reporting beneficial effects^20,27^, and others suggesting detrimental outcomes^25,26,64,65^. These inconsistencies may, in part, stem from a failure to differentiate among distinct facets of affective experience (e.g., high vs. low-arousal PA), as well as from variability in how weak ties were defined or operationalized across studies. Unlike previous studies that often focused narrowly on interactions with *strangers*, we defined “distant companions” more broadly to include coworkers and acquaintances, while explicitly distinguishing them from close companions. Within this framework, our findings suggest that the mood-enhancing effects of social engagement are strongest in interactions with close ties, though modest benefits are also evident in high-arousal PA during interactions with more distal social connections. These findings not only replicate prior work underscoring the importance of close companionship for emotional well-being but also clarify that high-arousal PA can be elevated even in the context of weaker social ties.

The findings highlight a specific link between individual differences in amygdala reactivity and the experience of high-arousal PA, but not low-arousal PA or NA, across social contexts. These results are largely consistent with prior research indicating that heightened amygdala reactivity is linked to greater sensitivity to social cues and increased vulnerability to emotional difficulties in social settings^11,12^. Our study extends this literature by clarifying which real-world social contexts and affective states are most relevant to individual differences in amygdala reactivity. Specifically, our findings suggest that individuals with heightened amygdala reactivity may be more susceptible to reductions in high-arousal PA in solitary contexts, where opportunities for social connection are limited. This pattern is consistent with findings by Yan et al. ^37^, who reported that high-arousal PA (e.g., joy, happiness) is more strongly associated with affiliation satisfaction than low-arousal PA (e.g., contentment, tranquility). Another study found that low-arousal PA is more closely associated with mastery-related activities^66^, suggesting a stronger link to a sense of personal worth. Expanding on this line of work, the present findings suggest that heightened levels of amygdala may amplify the differential sensitivity of high- vs. low-arousal PA to variations in social context. Importantly, although our analyses were guided by an a priori hypothesis focusing specifically on high-arousal PA, the observed interaction effect did not survive Bonferroni correction for multiple comparisons across the three affective outcomes. Accordingly, this finding should be interpreted with caution and replicated in future studies with larger, more adequately powered samples.

Unlike PA, levels of NA did not significantly vary across social contexts as a function of amygdala reactivity. Unlike the circumplex model of emotion^28^, which posits orthogonal valence and arousal dimensions, our EMA factor analysis did not yield distinct high- vs. low-arousal for NA factors – consistent with prior findings that, in everyday contexts, NA tends to coalesce into a general distress factor with limited arousal-based differentiation^67,68^. One possibility is that this, combined with the low average levels and limited variability of NA typically observed in non-clinical sample^69–71^, including ours (*M* = 1.56, *SD* = 0.62), may have reduced sensitivity to detect context-related effects. Another consideration is that, unlike prior EMA-fMRI studies linking amygdala reactivity to NA in the context of explicitly negative social events (e.g., rejection)^72^, our study focused on everyday social contexts varying in relationship closeness. It is possible that NA, unlike PA, does not meaningfully fluctuate with social intimacy alone unless the emotional valence of interaction is also taken into account. Indeed, findings from Rusu and colleagues suggest that while positive emotion is positively associated with relationship intimacy, negative emotion does not show a significant association^73^. Similarly, Taylor and colleagues found that negative affect was not significantly influence intimacy even within close relationships such as romantic couples^74^. To address these possibilities, future studies using event-contingent EMA protocols that capture a broader range of affective and social experiences are needed to replicate and extend the current findings.

Despite its novel insights, this study has several limitations. First, while it revealed associations between neural markers and emotional functioning across different social contexts, causality cannot be inferred. Future research should use longitudinal designs or experimental manipulations to determine whether amygdala reactivity prospectively *predicts* changes in emotional experiences in various social contexts. Second, while we categorized social contexts based on the physical presence of others and the closeness of relationships, emotional experiences may also depend on factors such as interaction duration, intensity, type, and communication medium. Future studies should explore these variables to provide a more nuanced understanding of how the specific aspects of social interactions shape affective experiences. Lastly, this study was conducted with a non-clinical sample. Research with clinical populations is needed to explicate how the relationships between neural markers, social context, and affect contribute to the development and maintenance of emotion-related disorders.

In summary, the present findings provide new insights regarding the relationship between amygdala reactivity, affective experiences, and social contexts. By integrating EMA and task-based fMRI data, this study enhances the translational relevance of these findings and provides a foundation for understanding the mechanisms underlying individual differences in emotional responses to varying social contexts.

## Supplementary Information

Below is the link to the electronic supplementary material.

Supplementary Material 1

## Data Availability

The processed data are publicly available on the Open Science Framework (OSF) website at https://doi.org/10.17605/osf.io/k2jdz. The analysis scripts are publicly available on the OSF website at https://doi.org/10.17605/osf.io/k2jdz.
