# Supplementary material for: The role of amygdala reactivity in affective fluctuations across social contexts
Source: Sci Rep. 2025 Oct 31;15:38198. doi: 10.1038/s41598-025-22131-x (PMC12578825; doi:10.1038/s41598-025-22131-x)
Supplement: Supplementary file 1 — Supplementary Material 1 [file 41598_2025_22131_MOESM1_ESM.pdf]

Supplementary Method and Results

Chae-eun Chung[1]

Hakin Kim[1]

Junhyun Park[1]

M.Justin Kim[2, 3]

Juyoen Hur[1]

[1] Department of Psychology, Yonsei University, Seoul 03722, Republic of Korea

[2] Department of Psychology, Sungkyunkwan University, Seoul 03063, Republic of Korea

[3] Center for Neuroscience Imaging Research, Institute for Basic Science, Suwon 16419, Republic of Korea

Address Correspondence to:

Juyoen Hur (jhur1@yonsei.ac.kr)

SUPPLEMENTARY METHOD

Subject screening

As part of an ongoing prospective-longitudinal study focused on identifying the biomarkers for internalizing disorders, we used well-established measures of neuroticism to screen 3,281 young Korean adults (75.7% female; M = 22.1 years, SD = 2.0 years). Screening data were stratified into three quartiles (top quartile, middle quartile, bottom quartile). Individuals who met preliminary inclusion criteria were independently recruited from each of the resulting three strata. Given the focus of the larger study, individuals in the high neuroticism group were slightly oversampled (i.e., 40% high, 30% middle, 30% bottom), enabling us to sample a wide range of individuals who are at risk for the development of internalizing disorders.

Neuroticism questionnaires

Three well-established questionnaires were used to measure neuroticism, including the neuroticism subscales from the NEO five-factor inventory [1,]12 items; [2], the Big-five inventory 12 items; [3], and the Behavioral Inhibition Scales 7 items; [4]. The standardized scores of each questionnaire were averaged to create a composite score of neuroticism. The composite scores captured a wide range of neuroticism (z = -1.87 ~ 1.93).

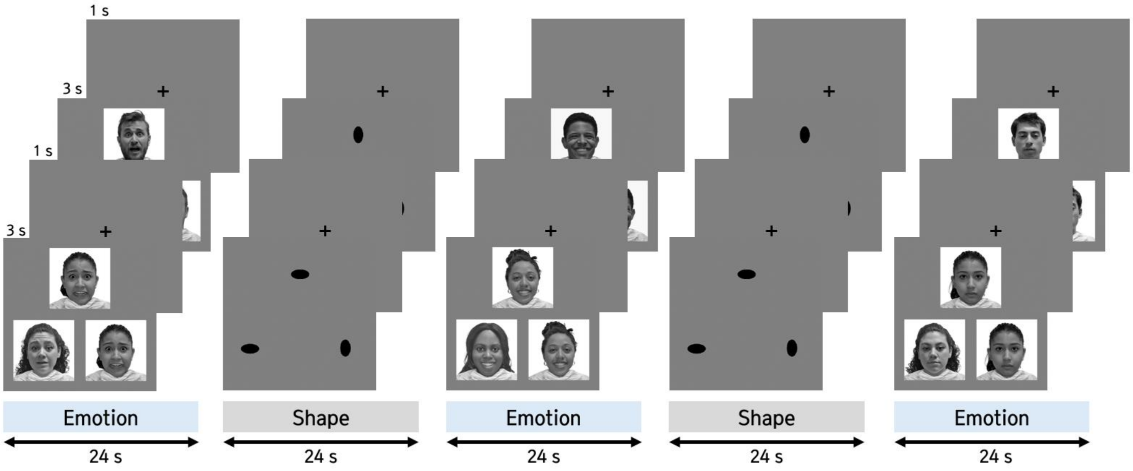

Fig. S1. Schematic of the Modified Face-Matching Task [5]

MRI data preprocessing

Anatomical data. T1-weighted images were inhomogeneity corrected using *N4* software [7], distributed with *Advanced Neuroimaging Tools* ANTs; [8]. Brain-extracted T1 images of cerebrospinal fluid, white-matter and gray-matter were segmented using *FAST* FSL 6.0.5; [9]. Brain surfaces were reconstructed using *FreeSurfer 7.2.0* [10], and the brain mask estimated previously was refined with a custom variation of the method to reconcile *ANTs*-derived and *FreeSurfer*-derived segmentations of the cortical gray-matter of *Mindboggle* [11]. Brain-extracted T1 images were normalized to the standard space MNI ICBM 152 non-linear 6th generation Asymmetric Average Brain Stereotaxic Registration Model MNI152NLin6Asym; [12], through nonlinear registration with *ANTs 2.3.3* [8].

Fieldmap data. The fieldmap images were estimated based on echo-planar imaging (EPI) references with *topup* software [9]. If an error was found in the method using *topup* software, the fieldmap images were estimated from the phase-drift maps measure with two consecutive gradient-recalled echo (GRE) acquisitions.

SUPPLEMENTARY RESULTS

	MR1	MR2	MR3	Category
Enthusiastic	0.09	-0.01	**0.86**	High-arousal PA
Joyful	-0.03	0.08	**0.94**	
Cheerful	-0.02	0.15	**0.84**	
Calm	-0.04	**0.95**	0.01	Low-arousal PA
Content	-0.04	**0.77**	0.24	
Relaxed	0.00	**0.98**	-0.01	
Nervous	**0.78**	-0.09	-0.01	NA
Worry	**0.79**	-0.06	-0.05	
Afraid	**0.87**	-0.10	0.08	
Irritable	**0.87**	0.06	-0.02	
Angry	**0.85**	-0.02	0.12	
Downhearted	**0.81**	0.18	-0.29	
Sad	**0.86**	-0.06	0.10	
Tired	**0.58**	0.01	-0.26	
Hopeless	**0.89**	-0.05	0.11	
Lonely	**0.67**	0.01	-0.08	

Table. S1. Exploratory factor analysis results. EFA yielded a three-factor solution at the between-person level. Factor 1 consisted of three high-arousal PA items (i.e., enthusiastic, joyful, cheerful), factor 2 consisted of three low-arousal PA items (i.e., calm, content, relaxed), and factor 3 consisted of all 10 NA items.

		t	*b*	*S.E.*	*p*
High-arousal PA	(Close vs. Alone)	7.03	0.26	0.04	< 0.001 ***
	(Close vs. Distant)	1.15	0.07	0.06	0.26
	(Distant vs. Alone)	3.44	0.19	0.05	0.001 **
Low-arousal PA	(Close vs. Alone)	4.36	0.15	0.04	< 0.001 ***
	(Close vs. Distant)	4.40	0.09	0.05	< 0.001 ***
	(Distant vs. Alone)	-1.60	-0.09	0.05	0.12
NA	(Close vs. Alone)	-3.65	-0.08	0.02	< 0.001 ***
	(Close vs. Distant)	-2.71	-0.09	0.03	0.008 **
	(Distant vs. Alone)	0.15	0.00	0.03	0.88

Table. S2. Momentary emotional experiences across different social contexts.

		t	*b*	*S.E.*	*p*
High-arousal PA	(Close vs. Alone)	7.01	0.26	0.04	< 0.001 ***
	(Distant vs. Alone)	3.38	0.19	0.05	0.001 **
Low-arousal PA	(Close vs. Alone)	4.29	0.15	0.04	< 0.001 ***
	(Close vs. Distant)	4.40	0.24	0.05	< 0.001 ***
NA	(Close vs. Alone)	-3.68	-0.08	0.02	< 0.001 ***
	(Close vs. Distant)	-2.79	-0.09	0.03	0.006 **

Table. S3. Momentary emotional experiences across different social contexts, controlling for age, sex and the amount of time allocated to social contexts. Note: control analyses were conducted only for statistically significant results.

	High-arousal PA			
	t	***b***	***S.E.***	***p***
**Amygdala**	-0.92	-0.23	0.25	0.36
**Close (vs. Alone)**	3.79	0.19	0.05	< 0.001 ***
**Distant (vs. Alone)**	1.92	0.14	0.07	0.06
**Amygdala × Close (vs. Alone)**	2.05	0.23	0.11	0.044 *
**Amygdala × Distant (vs. Alone)**	1.13	0.18	0.16	0.26

Table. S4. Association of amygdala reactivity with real-world emotional experiences in high-arousal PA, controlling for sex, age, neuroticism score, and the time allocated to social contexts. Note: even after including neuroticism score as a control variable, the results remained significant confirming the unique explanatory value of amygdala reactivity.

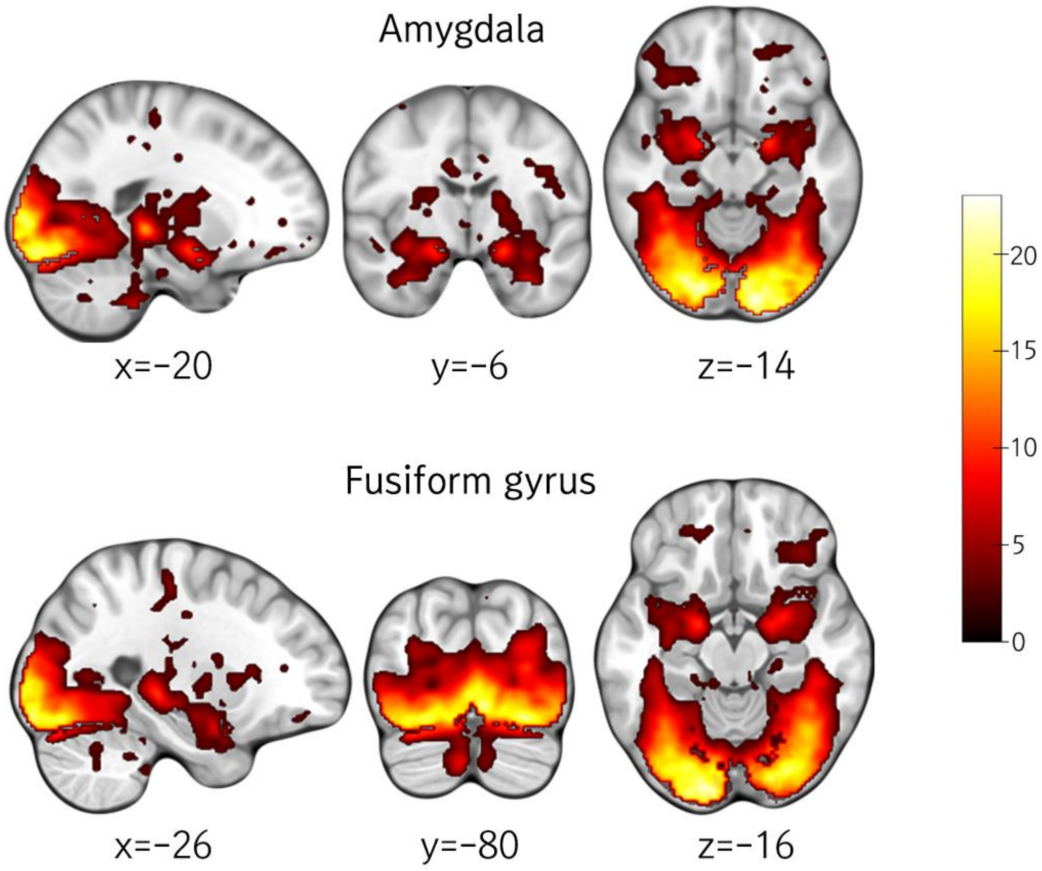

Figure. S2. fMRI task effects. Consistent with prior studies [13,14], the amygdala (peak x,y,z = -20, -6, -14) and fusiform gyrus (peak x,y,z = -26, -80, -16) showed significant activation in the fearful faces vs. shapes contrast. Brain images were generated using MRIcroGL software (version 1.2.20220720), available at https://www.nitrc.org/projects/mricrogl.

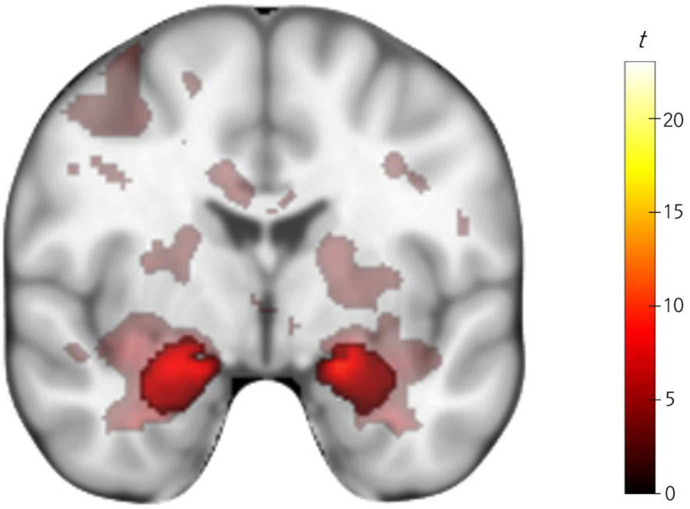

Figure. S3. Bilateral amygdala masks used for the integrative analyses of fMRI and EMA were highlighted in vivid red. Brain images were generated using MRIcroGL software (version 1.2.20220720), available at https://www.nitrc.org/projects/mricrogl.

REFERENCES

1. Costa, P. T. & McCrae, R. R. The revised neo personality inventory (neo-pi-r). *The SAGE handbook of personality theory and assessment* **2**, 179-198 (2008).
2. Costa, P. T. & McCrae, R. R. Normal personality assessment in clinical practice: The NEO Personality Inventory. *Psychological assessment* **4**, 5 (1992).
3. Soto, C. J. & John, O. P. The next Big Five Inventory (BFI-2): Developing and assessing a hierarchical model with 15 facets to enhance bandwidth, fidelity, and predictive power. *Journal of personality and social psychology* **113**, 117 (2017).
4. Gray, J. A. in *A model for personality* 246-276 (Springer, 1981).
5. Hariri, A. R., Tessitore, A., Mattay, V. S., Fera, F. & Weinberger, D. R. The amygdala response to emotional stimuli: a comparison of faces and scenes. *Neuroimage* **17**, 317-323 (2002).
6. Conley, M. I. *et al.* The racially diverse affective expression (RADIATE) face stimulus set. *Psychiatry research* **270**, 1059-1067 (2018).
7. Tustison, N. J. *et al.* N4ITK: improved N3 bias correction. *IEEE transactions on medical imaging* **29**, 1310-1320 (2010).
8. Avants, B. B. *et al.* A reproducible evaluation of ANTs similarity metric performance in brain image registration. *Neuroimage* **54**, 2033-2044 (2011).
9. Jenkinson, M., Beckmann, C. F., Behrens, T. E., Woolrich, M. W. & Smith, S. M. Fsl. *Neuroimage* **62**, 782-790 (2012).
10. Dale, A. M., Fischl, B. & Sereno, M. I. Cortical surface-based analysis: I. Segmentation and surface reconstruction. *Neuroimage* **9**, 179-194 (1999).
11. Klein, A. *et al.* Mindboggling morphometry of human brains. *PLoS computational biology* **13**, e1005350 (2017).
12. Evans, A. C., Janke, A. L., Collins, D. L. & Baillet, S. Brain templates and atlases. *Neuroimage* **62**, 911-922 (2012).
13. Hur, J. *et al.* Anxiety-related frontocortical activity is associated with dampened stressor

reactivity in the real world. *Psychological Science* **33**, 906-924 (2022).

14. Swartz, J. R., Knodt, A. R., Radtke, S. R. & Hariri, A. R. A neural biomarker of psychological vulnerability to future life stress. *Neuron* **85**, 505-511 (2015).

Standardized report for fMRIPrep

Results included in this manuscript come from preprocessing performed using *fMRIPrep* 22.1.1 (Esteban, Markiewicz, et al. (2018); Esteban, Blair, et al. (2018); RRID:SCR_016216), which is based on *Nipype* 1.8.5 (K. Gorgolewski et al. (2011); K. J. Gorgolewski et al. (2018); RRID:SCR_002502).

Preprocessing of B_0 inhomogeneity mappings

A total of 4 fieldmaps were found available within the input BIDS structure for this particular subject. A *B_0*-nonuniformity map (or *fieldmap*) was estimated based on two (or more) echo-planar imaging (EPI) references with topup (Andersson, Skare, and Ashburner (2003); FSL 6.0.5.1:57b01774).

Anatomical data preprocessing

A total of 1 T1-weighted (T1w) images were found within the input BIDS dataset.The T1-weighted (T1w) image was corrected for intensity non-uniformity (INU) with N4BiasFieldCorrection (Tustison et al. 2010), distributed with ANTs 2.3.3 (Avants et al. 2008, RRID:SCR_004757), and used as T1w-reference throughout the workflow. The T1w-reference was then skull-stripped with a *Nipype* implementation of the antsBrainExtraction.sh workflow (from ANTs), using OASIS30ANTs as target template. Brain tissue segmentation of cerebrospinal fluid (CSF), white-matter (WM) and gray-matter (GM) was performed on the brain-extracted T1w using fast (FSL 6.0.5.1:57b01774, RRID:SCR_002823, Zhang, Brady, and Smith 2001). Brain surfaces were reconstructed using recon-all (FreeSurfer 7.2.0, RRID:SCR_001847, Dale, Fischl, and Sereno 1999), and the brain mask estimated previously was refined with a custom variation of the method to reconcile ANTs-derived and FreeSurfer-derived segmentations of the cortical gray-matter of Mindboggle (RRID:SCR_002438, Klein et al. 2017). Volume-based spatial normalization to two standard spaces (MNI152NLin2009cAsym, MNI152NLin6Asym) was performed through nonlinear registration with antsRegistration (ANTs 2.3.3), using brain-extracted versions of both T1w reference and the T1w template. The following templates were selected for spatial normalization: *ICBM 152 Nonlinear Asymmetrical template version 2009c* [Fonov et al. (2009), RRID:SCR_008796; TemplateFlow ID: MNI152NLin2009cAsym], *FSL's MNI ICBM 152 non-linear 6th Generation Asymmetric Average Brain Stereotaxic Registration Model* [Evans et al. (2012), RRID:SCR_002823; TemplateFlow ID: MNI152NLin6Asym].

Functional data preprocessing

For each of the 1 BOLD runs found per subject (across all tasks and sessions), the following preprocessing was performed. First, a reference volume and its skull-stripped version were generated using a custom methodology of *fMRIPrep*. Head-motion parameters with respect to the BOLD reference (transformation matrices, and six corresponding rotation and translation parameters) are estimated before any spatiotemporal filtering using mcflirt (FSL 6.0.5.1:57b01774, Jenkinson et al. 2002). The estimated *fieldmap* was then aligned with rigid-registration to the target EPI (echo-planar imaging) reference run. The field coefficients were mapped on to the reference EPI using the transform. BOLD runs were slice-time corrected to 0.708s (0.5 of slice acquisition range 0s-1.42s) using 3dTshift from AFNI (Cox and Hyde 1997, RRID:SCR_005927). The BOLD reference was then co-registered to the T1w reference using bbregister (FreeSurfer) which implements boundary-based registration (Greve and Fischl 2009). Co-registration was configured with six degrees of freedom. Several confounding time-series were calculated based on the *preprocessed BOLD*: framewise displacement (FD), DVARS and three region-wise global signals. FD was computed using two formulations following Power (absolute sum of relative motions, Power et al. (2014)) and Jenkinson (relative root mean square displacement between affines, Jenkinson et al. (2002)). FD and DVARS are calculated for each functional run, both using their implementations in *Nipype* (following the definitions by Power et al. 2014). The three global signals are extracted within the CSF, the WM, and the whole-brain masks. Additionally, a set of physiological regressors were extracted to allow for component-based noise correction (*CompCor*, Behzadi et al. 2007). Principal components are estimated after high-pass filtering the *preprocessed BOLD* time-series (using a discrete cosine filter with 128s cut-off) for the two *CompCor* variants: temporal (tCompCor) and anatomical (aCompCor). tCompCor components are then calculated from the top 2% variable voxels within the brain mask. For aCompCor, three probabilistic masks (CSF, WM and combined CSF+WM) are generated in anatomical space. The implementation differs from that of Behzadi et al. in that instead of eroding the masks by 2 pixels on BOLD space, a mask of pixels that likely contain a volume fraction of GM is subtracted from the aCompCor masks. This mask is obtained by dilating a GM mask extracted from the FreeSurfer's *aseg* segmentation, and it ensures components are not extracted from voxels containing a minimal fraction of GM. Finally, these masks are resampled into BOLD space and binarized by thresholding at 0.99 (as in the original implementation). Components are also calculated separately within the WM and CSF masks. For each CompCor decomposition, the *k* components with the largest singular values are retained, such that the retained components' time series are sufficient to explain 50 percent of variance across the nuisance mask (CSF, WM, combined, or temporal). The remaining components are

dropped from consideration. The head-motion estimates calculated in the correction step were also placed within the corresponding confounds file. The confound time series derived from head motion estimates and global signals were expanded with the inclusion of temporal derivatives and quadratic terms for each (Satterthwaite et al. 2013). Frames that exceeded a threshold of 0.5 mm FD or 1.5 standardized DVARS were annotated as motion outliers. Additional nuisance timeseries are calculated by means of principal components analysis of the signal found within a thin band (*crown*) of voxels around the edge of the brain, as proposed by (Patriat, Reynolds, and Birn 2017). The BOLD time-series were resampled into standard space, generating a *preprocessed BOLD run in MNI152NLin2009cAsym space*. First, a reference volume and its skull-stripped version were generated using a custom methodology of *fMRIPrep*. Automatic removal of motion artifacts using independent component analysis (ICA-AROMA, Pruim et al. 2015) was performed on the *preprocessed BOLD on MNI space* time-series after removal of non-steady state volumes and spatial smoothing with an isotropic, Gaussian kernel of 6mm FWHM (full-width half-maximum). Corresponding "non-aggresively" denoised runs were produced after such smoothing. Additionally, the "aggressive" noise-regressors were collected and placed in the corresponding confounds file. All resamplings can be performed with *a single interpolation step* by composing all the pertinent transformations (i.e. head-motion transform matrices, susceptibility distortion correction when available, and co-registrations to anatomical and output spaces). Gridded (volumetric) resamplings were performed using antsApplyTransforms (ANTs), configured with Lanczos interpolation to minimize the smoothing effects of other kernels (Lanczos 1964). Non-gridded (surface) resamplings were performed using mri_vol2surf (FreeSurfer).

Functional data preprocessing

For each of the 5 BOLD runs found per subject (across all tasks and sessions), the following preprocessing was performed. First, a reference volume and its skull-stripped version were generated using a custom methodology of *fMRIPrep*. Head-motion parameters with respect to the BOLD reference (transformation matrices, and six corresponding rotation and translation parameters) are estimated before any spatiotemporal filtering using mcflirt (FSL 6.0.5.1:57b01774, Jenkinson et al. 2002). BOLD runs were slice-time corrected to 0.708s (0.5 of slice acquisition range 0s-1.42s) using 3dTshift from AFNI (Cox and Hyde 1997, RRID:SCR_005927). The BOLD time-series (including slice-timing correction when applied) were resampled onto their original, native space by applying the transforms to correct for head-motion. These resampled BOLD time-series will be referred to as *preprocessed BOLD in original space*, or just *preprocessed BOLD*. The BOLD reference was then co-registered to the T1w

reference using bbregister (FreeSurfer) which implements boundary-based registration (Greve and Fischl 2009). Co-registration was configured with six degrees of freedom. Several confounding time-series were calculated based on the *preprocessed BOLD*: framewise displacement (FD), DVARS and three region-wise global signals. FD was computed using two formulations following Power (absolute sum of relative motions, Power et al. (2014)) and Jenkinson (relative root mean square displacement between affines, Jenkinson et al. (2002)). FD and DVARS are calculated for each functional run, both using their implementations in *Nipype* (following the definitions by Power et al. 2014). The three global signals are extracted within the CSF, the WM, and the whole-brain masks. Additionally, a set of physiological regressors were extracted to allow for component-based noise correction (*CompCor*, Behzadi et al. 2007). Principal components are estimated after high-pass filtering the *preprocessed BOLD* time-series (using a discrete cosine filter with 128s cut-off) for the two *CompCor* variants: temporal (tCompCor) and anatomical (aCompCor). tCompCor components are then calculated from the top 2% variable voxels within the brain mask. For aCompCor, three probabilistic masks (CSF, WM and combined CSF+WM) are generated in anatomical space. The implementation differs from that of Behzadi et al. in that instead of eroding the masks by 2 pixels on BOLD space, a mask of pixels that likely contain a volume fraction of GM is subtracted from the aCompCor masks. This mask is obtained by dilating a GM mask extracted from the FreeSurfer's *aseg* segmentation, and it ensures components are not extracted from voxels containing a minimal fraction of GM. Finally, these masks are resampled into BOLD space and binarized by thresholding at 0.99 (as in the original implementation). Components are also calculated separately within the WM and CSF masks. For each CompCor decomposition, the *k* components with the largest singular values are retained, such that the retained components' time series are sufficient to explain 50 percent of variance across the nuisance mask (CSF, WM, combined, or temporal). The remaining components are dropped from consideration. The head-motion estimates calculated in the correction step were also placed within the corresponding confounds file. The confound time series derived from head motion estimates and global signals were expanded with the inclusion of temporal derivatives and quadratic terms for each (Satterthwaite et al. 2013). Frames that exceeded a threshold of 0.5 mm FD or 1.5 standardized DVARS were annotated as motion outliers. Additional nuisance timeseries are calculated by means of principal components analysis of the signal found within a thin band (*crown*) of voxels around the edge of the brain, as proposed by (Patriat, Reynolds, and Birn 2017). The BOLD time-series were resampled into standard space, generating a *preprocessed BOLD run in MNI152NLin2009cAsym space*. First, a reference volume and its

skull-stripped version were generated using a custom methodology of *fMRIPrep*. Automatic removal of motion artifacts using independent component analysis (ICA-AROMA, Pruim et al. 2015) was performed on the *preprocessed BOLD on MNI space* time-series after removal of non-steady state volumes and spatial smoothing with an isotropic, Gaussian kernel of 6mm FWHM (full-width half-maximum). Corresponding "non-aggresively" denoised runs were produced after such smoothing. Additionally, the "aggressive" noise-regressors were collected and placed in the corresponding confounds file. All resamplings can be performed with *a single interpolation step* by composing all the pertinent transformations (i.e. head-motion transform matrices, susceptibility distortion correction when available, and co-registrations to anatomical and output spaces). Gridded (volumetric) resamplings were performed using antsApplyTransforms (ANTs), configured with Lanczos interpolation to minimize the smoothing effects of other kernels (Lanczos 1964). Non-gridded (surface) resamplings were performed using mri_vol2surf (FreeSurfer).

Many internal operations of *fMRIPrep* use *Nilearn* 0.9.1 (Abraham et al. 2014, RRID:SCR_001362), mostly within the functional processing workflow. For more details of the pipeline, see the section corresponding to workflows in *fMRIPrep*'s documentation.

Copyright Waiver

The above boilerplate text was automatically generated by fMRIPrep with the express intention that users should copy and paste this text into their manuscripts *unchanged*. It is released under the CC0 license.

References

Abraham, Alexandre, Fabian Pedregosa, Michael Eickenberg, Philippe Gervais, Andreas Mueller, Jean Kossaifi, Alexandre Gramfort, Bertrand Thirion, and Gael Varoquaux. 2014. "Machine Learning for Neuroimaging with Scikit-Learn." *Frontiers in Neuroinformatics* 8. https://doi.org/10.3389/fninf.2014.00014.

Andersson, Jesper L. R., Stefan Skare, and John Ashburner. 2003. "How to Correct Susceptibility Distortions in Spin-Echo Echo-Planar Images: Application to Diffusion Tensor Imaging." *NeuroImage* 20 (2): 870–88. https://doi.org/10.1016/S1053-8119(03)00336-7.

Avants, B. B., C. L. Epstein, M. Grossman, and J. C. Gee. 2008. "Symmetric Diffeomorphic Image Registration with Cross-Correlation: Evaluating Automated Labeling of Elderly and Neurodegenerative Brain." *Medical Image Analysis* 12 (1): 26–

41. https://doi.org/10.1016/j.media.2007.06.004.

Behzadi, Yashar, Khaled Restom, Joy Liau, and Thomas T. Liu. 2007. "A Component Based Noise Correction Method (CompCor) for BOLD and Perfusion Based fMRI." *NeuroImage* 37 (1): 90–101. https://doi.org/10.1016/j.neuroimage.2007.04.042.

Cox, Robert W., and James S. Hyde. 1997. "Software Tools for Analysis and Visualization of fMRI Data." *NMR in Biomedicine* 10 (4-5): 171–78. https://doi.org/10.1002/(SICI)1099-1492(199706/08)10:4/5<171::AID-NBM453>3.0.CO;2-L.

Dale, Anders M., Bruce Fischl, and Martin I. Sereno. 1999. "Cortical Surface-Based Analysis: I. Segmentation and Surface Reconstruction." *NeuroImage* 9 (2): 179–94. https://doi.org/10.1006/nimg.1998.0395.

Esteban, Oscar, Ross Blair, Christopher J. Markiewicz, Shoshana L. Berleant, Craig Moodie, Feilong Ma, Ayse Ilkay Isik, et al. 2018. "fMRIPrep 22.1.1." *Software*. https://doi.org/10.5281/zenodo.852659.

Esteban, Oscar, Christopher Markiewicz, Ross W Blair, Craig Moodie, Ayse Ilkay Isik, Asier Erramuzpe Aliaga, James Kent, et al. 2018. "fMRIPrep: A Robust Preprocessing Pipeline for Functional MRI." *Nature Methods*. https://doi.org/10.1038/s41592-018-0235-4.

Evans, AC, AL Janke, DL Collins, and S Baillet. 2012. "Brain Templates and Atlases." *NeuroImage* 62 (2): 911–22. https://doi.org/10.1016/j.neuroimage.2012.01.024.

Fonov, VS, AC Evans, RC McKinstry, CR Almli, and DL Collins. 2009. "Unbiased Nonlinear Average Age-Appropriate Brain Templates from Birth to Adulthood." *NeuroImage* 47, Supplement 1: S102. https://doi.org/10.1016/S1053-8119(09)70884-5.

Gorgolewski, K., C. D. Burns, C. Madison, D. Clark, Y. O. Halchenko, M. L. Waskom, and S. Ghosh. 2011. "Nipype: A Flexible, Lightweight and Extensible Neuroimaging Data Processing Framework in Python." *Frontiers in Neuroinformatics* 5: 13. https://doi.org/10.3389/fninf.2011.00013.

Gorgolewski, Krzysztof J., Oscar Esteban, Christopher J. Markiewicz, Erik Ziegler, David Gage Ellis, Michael Philipp Notter, Dorota Jarecka, et al. 2018. "Nipype." *Software*. https://doi.org/10.5281/zenodo.596855.

Greve, Douglas N, and Bruce Fischl. 2009. "Accurate and Robust Brain Image Alignment Using Boundary-Based Registration." *NeuroImage* 48 (1): 63–72. https://doi.org/10.1016/j.neuroimage.2009.06.060.

Jenkinson, Mark, Peter Bannister, Michael Brady, and Stephen Smith. 2002. “Improved Optimization for the Robust and Accurate Linear Registration and Motion Correction of Brain Images.” *NeuroImage* 17 (2): 825–41. https://doi.org/10.1006/nimg.2002.1132.

Klein, Arno, Satrajit S. Ghosh, Forrest S. Bao, Joachim Giard, Yrjö Häme, Eliezer Stavsky, Noah Lee, et al. 2017. “Mindboggling Morphometry of Human Brains.” *PLOS Computational Biology* 13 (2): e1005350. https://doi.org/10.1371/journal.pcbi.1005350.

Lanczos, C. 1964. “Evaluation of Noisy Data.” *Journal of the Society for Industrial and Applied Mathematics Series B Numerical Analysis* 1 (1): 76–85. https://doi.org/10.1137/0701007.

Patriat, Rémi, Richard C. Reynolds, and Rasmus M. Birn. 2017. “An Improved Model of Motion-Related Signal Changes in fMRI.” *NeuroImage* 144, Part A (January): 74–82. https://doi.org/10.1016/j.neuroimage.2016.08.051.

Power, Jonathan D., Anish Mitra, Timothy O. Laumann, Abraham Z. Snyder, Bradley L. Schlaggar, and Steven E. Petersen. 2014. “Methods to Detect, Characterize, and Remove Motion Artifact in Resting State fMRI.” *NeuroImage* 84 (Supplement C): 320–41. https://doi.org/10.1016/j.neuroimage.2013.08.048.

Pruim, Raimon H. R., Maarten Mennes, Daan van Rooij, Alberto Llera, Jan K. Buitelaar, and Christian F. Beckmann. 2015. “ICA-AROMA: A Robust ICA-Based Strategy for Removing Motion Artifacts from fMRI Data.” *NeuroImage* 112 (Supplement C): 267–77. https://doi.org/10.1016/j.neuroimage.2015.02.064.

Satterthwaite, Theodore D., Mark A. Elliott, Raphael T. Gerraty, Kosha Ruparel, James Loughead, Monica E. Calkins, Simon B. Eickhoff, et al. 2013. “An improved framework for confound regression and filtering for control of motion artifact in the preprocessing of resting-state functional connectivity data.” *NeuroImage* 64 (1): 240–56. https://doi.org/10.1016/j.neuroimage.2012.08.052.

Tustison, N. J., B. B. Avants, P. A. Cook, Y. Zheng, A. Egan, P. A. Yushkevich, and J. C. Gee. 2010. “N4itk: Improved N3 Bias Correction.” *IEEE Transactions on Medical Imaging* 29 (6): 1310–20. https://doi.org/10.1109/TMI.2010.2046908.

Zhang, Y., M. Brady, and S. Smith. 2001. “Segmentation of Brain MR Images Through a Hidden Markov Random Field Model and the Expectation-Maximization Algorithm.” *IEEE Transactions on Medical Imaging* 20 (1): 45–57. https://doi.org/10.1109/42.906424.
